# Potential associated factors of functional disability in Chinese older inpatients: a multicenter cross-sectional study

**DOI:** 10.1186/s12877-020-01738-x

**Published:** 2020-09-03

**Authors:** Hongpeng Liu, Jing Jiao, Chen Zhu, Minglei Zhu, Xianxiu Wen, Jingfen Jin, Hui Wang, Dongmei Lv, Shengxiu Zhao, Xinjuan Wu, Tao Xu

**Affiliations:** 1 Department of Nursing, Chinese Academy of Medical Sciences - Peking Union Medical College, Peking Union Medical College Hospital (Dongdan campus), No.1 Shuaifuyuan Wangfujing Dongcheng District, Beijing, 100730 China; 2 Department of Geriatrics, Chinese Academy of Medical Sciences - Peking Union Medical College, Peking Union Medical College Hospital (Dongdan campus), No.1 Shuaifuyuan Wangfujing Dongcheng District, Beijing, 100730 China; 3 Department of Nursing, Sichuan Provincial People’s Hospital, No.32 West Second Section First Ring Road, Chengdu, 610072 China; 4 Department of Nursing, The Second Affiliated Hospital Zhejiang University School of Medicine, 88 Jiefang Road, Hangzhou, 310009 China; 5 Department of Nursing, Tongji Hospital, Tongji medical college, Huazhong University of Science and Technology, 1037 Luoyu Road, Hongshan District, Wuhan, 430074 China; 6 Department of Nursing, The Second Affiliated Hospital of Haerbin medical University, 246 Xuefu Road, Haerbin, 150081 China; 7 Department of Nursing, Qinghai Provincial People’s Hospital, 2 Gonghe Road, Chengdong District, Xining, 810007 China; 8 Department of Epidemiology and Statistics, Institute of Basic Medical Sciences, Chinese Academy of Medical Sciences & School of Basic Medicine, Peking Union Medical College, 5 Dongdan Santiao, Dongcheng District, Beijing, 100005 China

**Keywords:** Aging, Activities of daily living, Instrumental activities of daily living, Functional disability, Older inpatients, Chinese

## Abstract

**Background:**

There is still controversy about the relationship between aging and changes in functional ability. This study aims to describe the level of basic activities of daily living (ADL) and higher-level instrumental activities of daily living (IADL) in different age groups and explore the factors associated with functional disability in Chinese older inpatients.

**Methods:**

This cross-sectional study surveyed 9996 older inpatients aged 65 years and older from six tertiary hospitals in China from October 2018 to February 2019. The levels of ADL and IADL were measured by scores of the Barthel index and Instrumental Activities of Daily Living Scale. A mixed-effect generalized linear model was used to examine the association between functional disability and covariates.

**Results:**

The average ADL score was 89.51 ± 19.29 and the mean IADL score 6.76 ± 2.01 for all participants. There was a trend of decreasing scores along with aging, and significant differences between age groups were also observed (*P* < 0.001). The most affected ADL and IADL was stair climbing and shopping, respectively. Sociodemographic characteristics (such as age), physical health variables (frailty, emaciation, hearing dysfunction, urinary dysfunction, defecation dysfunction, falling accidents in the past 12 months), and mental health variables (cognitive dysfunction, depression) were associated with functional disability. Patients from the emergency department or transferred from other hospitals and former alcohol drinkers are at risk of ADL disability. Former smoking is a risk factor for IADL disability, whereas current drinking, higher-level education, and residing in a building without elevators were likely to maintain a better IADL performance.

**Conclusions:**

Functional ability declines with aging, older inpatients are low dependency upon ADL and IADL. There are several associated factors among the participants derived from this investigation of a large-scale, multicenter, nationally representative Chinese older inpatient population. These findings potentially have major importance for the planning of hospital services, discharge planning, and post-discharge care.

**Trial registration:**

Chinese Clinical Trial Registry, ChiCTR1800017682, registered August 9, 2018.

## Background

The aging population is increasing at an unprecedented rate worldwide [1]. The proportion of people aged 60 years and older is expected to double from about 11% in 2000 to 22% in 2050, with 80% living in low- to middle-income countries [2]. In 2018, the number of Chinese older adults approached 241 million, accounting for 17.2% of the total population, and this figure is expected to approach 480 million by 2050 [3, 4].

Older adults are likely to suffer from poor quality of life and irreversible decline in functional ability [1, 4], as they are vulnerable to a decline in physical functioning and find themselves unable to undertake the basic activities of daily living (ADL) and higher-level tasks that they used to [3]. Functional ability is defined in terms of higher-level instrumental activities of daily living (IADL) and basic ADL, including ability to use a telephone and personal items, feeding, bathing, grooming, and using public transport by oneself [5–7]. With the advent of the increasingly aging society, functional disability of older adults is a pressing concern for China and the world.

In 2017, Ran et al. indicated that a significant difference in functional ability exists among different age groups, the ADL ability in the 75 years age-group among Chinese Yi ethnic elderly did not abide by the age rule, which better than the 70 years age-group [8]. A Japanese study also suggested that the elderly in 80 years old group were significant differences in many items of ADL [9]. Besides, ADL and IADL measure different aspects of functional ability, while few studies on functional disability in China have focused on both ADL and IADL in older inpatients. Therefore, a comprehensive understanding of the level of ADL and IADL in different age groups among the older inpatients will contribute towards future health service planning.

Results from previous studies have varied with respect to the association between functional disability and potential associated factors, such as alcohol consumption, smoking, and body mass index (BMI) [10, 11]. However, factors affecting ADL and IADL among the elderly in different countries may differ. Also, previous estimates of functional disability in Chinese older inpatients used data from a smaller sample size [8]. To address this issue, we designed and conducted a study to explore the possible correlation between functional disability and age, and to examine the potential associated risk factors of functional disability in Chinese older inpatients based on a large-scale, cross-sectional national survey.

## Methods

### Study design and participants

Participants were derived from a large-scale cohort study, and the sample comes from a representative sample of the Chinese elderly inpatients in tertiary hospitals, which is an ongoing survey of physiological and psychological conditions in elder patients across the country (Chinese Clinical Trial Registry Number: ChiCTR1800017682) [12]. The baseline data collected from October 2018 to February 2019 represents the baseline survey data used in this study.

In China, the hospital level is divided into three levels. Large-scale national, provincial, and municipal tertiary hospitals have more than 500 beds and can provide complex medical care services, whereas secondary county and district hospitals usually have 100–499 beds, which provide basic specialty care and inpatient services, while community hospitals provide preventive and primary care services with less than 100 beds [13, 14]. The target population is all older patients in tertiary hospitals. In order to ensure the representativeness of the study sample, this study uses a two-stage cluster sampling method to recruit participants. For one thing, a simple random sampling method was used to select five provinces and one municipality in China (southwest: Sichuan province; northeast: Heilongjiang province; south-central: Hubei province; northern: Beijing municipality/city; northwest: Qinghai province; eastern: Zhejiang province). For another thing, a convenience sampling method and a simple random sampling method were used to select one tertiary hospital in each province or municipality/city, details of the sampling methods were described previously [12]. All eligible participants from the surgical, intensive care unit (ICU), neurology department, orthopedics department, and internal medicine of the selected hospitals were continuously recruited.

When the predicted prevalence rate is 4.9% [8], a sample size of 7161 can produce a two-sided 95% confidence interval with a tolerance error equal to 0.005. Taking into account the potential non-response and loss to follow, 10,000 subjects will be enrolled in this study. Inclusion criteria comprised 65 years of age and older; signed a consent form; understood the purpose of the study; and have sufficient mental ability to answer the interview questionnaire.

### Measurement instruments

ADL was evaluated by using the Barthel Index (BI), which is a 10-item instrument measuring disability in terms of a person’s level of functional independence in personal ADL. The Maryland State Medical Society holds the copyright for the Barthel Index. It may be used freely for noncommercial purposes with the following citation: Mahoney FI, Barthel D. “Functional evaluation: the Barthel Index.” Maryland State Med Journal 1965; 14:56–61. Used with permission [15, 16]. BI consists of 10 ADL, each of which is graded as 0, 5, or 10 with a maximum total score of 100 [17]. The larger score means the better capacity to perform daily living activities [16, 17].

The Instrumental Activities of Daily Living Scale (IADL) was used to measure IADL [5], which includes a series of higher-level activities that are considered to address the older adult’s capacity to interact with his or her community [18]. The scores of this scale range from 0 to 8. In the eight-item scale, 0 is the least independent, and 8 is the most independent [5, 8, 19].

### Data collection and quality control

The data were collected by trained and certified registered nurses. To ensure data quality, the research group developed the project survey manual, operation manual, and training manual. To guarantee the accuracy and integration of the data, the electronic data collection system (EDC system) was designed scientifically. To ensure accurate data collection, a total of 589 nurses received systematic training before they recorded patients’ information on the web-based online CRF. They are all proficient in the process of investigation, using the EDC system, and health assessment scale applications. Nurses collected baseline data such as demographic factors and physical and psychological conditions through face-to-face questionnaire interviews, physical examinations, clinical records, and health assessment. All CRFs were reviewed by the attentive head nurse in each ward to ensure the completeness and correctness of the raw data. Our research group also established a strict quality control team, and a communication platform based on the WeChat App in order to guarantee timely feedback. Details of the quality control team and communication platform were described previously [12].

### Definition of covariates

Potential factors associated with functional disability in this model included sex, marital status, vision, BMI, cognitive function, falling accidents in the past 12 months, education level, frailty, age, depression, admission to hospital, ethnicity, smoking, hearing, sleeping, urinary function, alcohol consumption, living conditions, and defecation function. BMI was measured in kg/m^2^ [20, 21]. Frailty was measured by the Frailty Scale [22], with a higher total score denoting more severe frail condition. Assessment of cognitive function was based on the Mini-Mental State Examination Scale [23], which was dichotomized as normal cognitive function and cognitive dysfunction. The depression assessment scale was based on the Geriatric Depression Scale 15 (GDS15) [24], with a larger GDS score meaning more severe depression.

### Statistical analysis

Continuous variables were described as means and standard deviations (SDs). Categorical variables were described as numbers and percentages. ANOVA was used to examine the statistical differences of variables among different age groups. Considering that the participants in the same ward or hospital were more likely to be assessed as having similar ADL or IADL scores, a mixed-effect generalized linear model with the hospital as a random effect was used to examine the relationship between functional disability and covariates in order to control the cluster effect of hospital wards. The regression coefficient and its 95% confidence interval (CI) were used to assess the strength of relationships. All statistical analyses were conducted using SAS 9.4 software (SAS Institute Inc., Cary, NC, USA). A *P* value of less than 0.05 was considered as statistical significance.

## Results

### Baseline demographic and clinical characteristics

Descriptive statistics and frequencies of demographics are shown in Supplementary File 1. A total of 9996 participants from 314 wards of six hospitals were enrolled in this study, there were 5778 males (57.80%) and 4218 females (42.20%). The mean age of this population was (72.47 ± 5.77) years old. Among them, 57.64% of participants were older than 70 years and 12.19% older than 80 years. Only 16.39% of the enrolled participants were illiterate. Approximately 88.81% of the participants were married. 94.16% of participants were of Han nationality. Normal BMI was measured in 48.54% of participants, and overweight patients accounted for the second biggest proportion in this regard (34.31%). More than 80% of participants were not in a frail condition although, notably, 82.28% of inpatients had symptoms of depression. An estimated one-third of participants had a smoking history and nearly one-quarter of a history of drinking alcohol. At least one falling accident in the previous 12 months was recorded in 14.23% of participants. In addition, other physical health variables including urinary dysfunction (14.11%), hearing dysfunction (19.40%), and mental health variables, such as cognitive dysfunction (20.57%) were present. With regard to living conditions, 16.95% of participants lived in a bungalow and the remainder lived in a building with (36.09%) or without (46.96%) an elevator.

### The level of ADL and IADL in different age groups

**Fig. 1:**
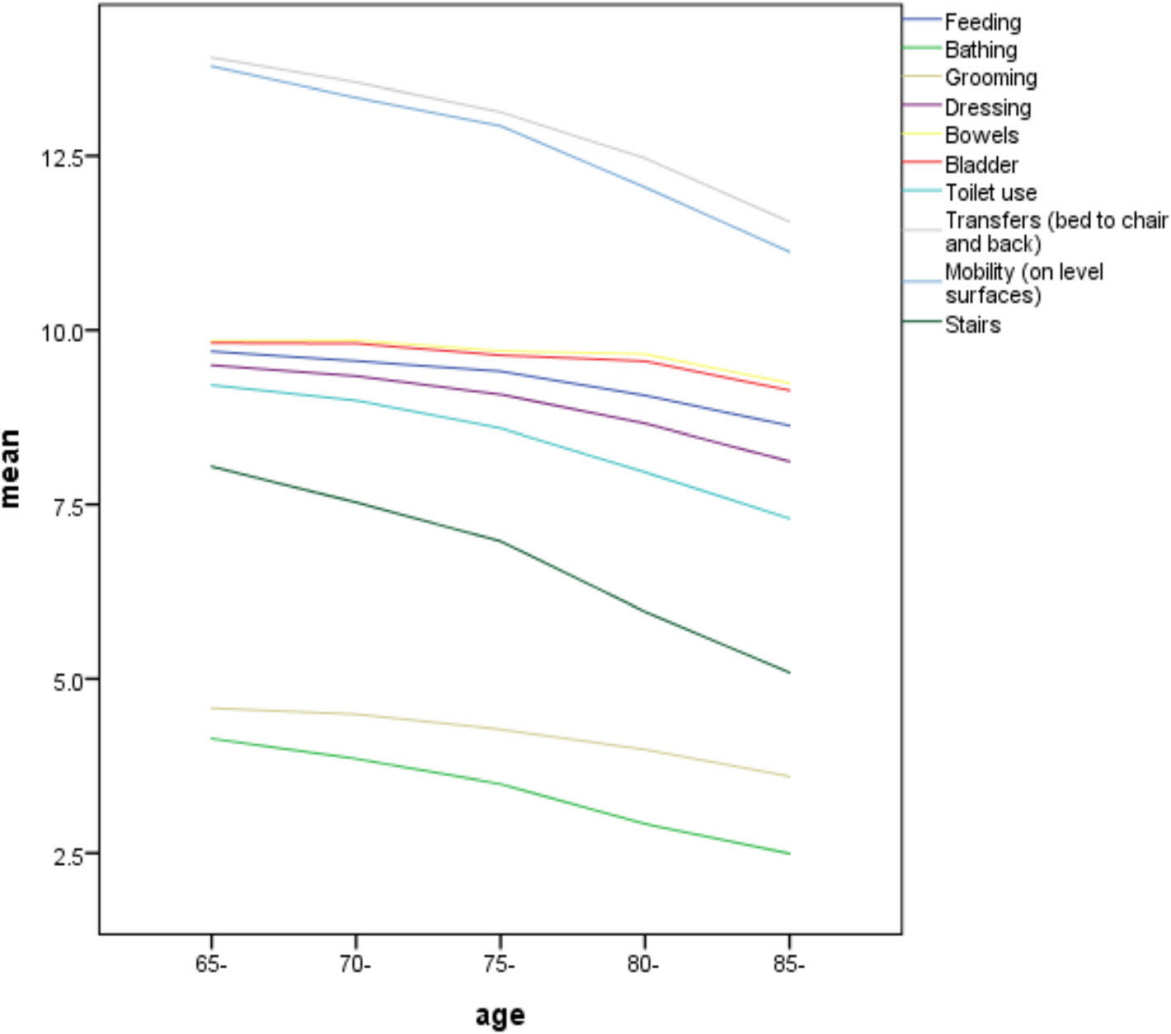
Risk of ADL by age. Abbreviations, ADL, basic activities of daily living

The levels of ADL and IADL among different age groups are presented in Table 1. The average ADL score was 89.51 ± 19.29 for all participants. The most affected ADL were walking up and down stairs, mobility (on level surfaces), transfers (bed to chair and back), toilet use, and bathing (see Fig. 1). In addition, there was a trend of decreasing scores along with aging, and significant differences between age groups were also observed (*P* < 0.001).

**Table 1:** One-way ANOVA of ADL and IADL scores among different age groups

Age-group (year)	ADL (mean ± SD)	IADL (mean ± SD)
65–69	92.51 ± 16.37	7.10 ± 1.77
70–74	90.30 ± 18.18	6.88 ± 1.88
75–79	87.20 ± 20.71	6.50 ± 2.16
80–84	82.27 ± 23.90	6.02 ± 2.31
85 and above	76.27 ± 27.42	4.96 ± 2.57
Total	89.51 ± 19.29	6.76 ± 2.01
*P* value	< 0.001	< 0.001

Abbreviations, ADL, basic activities of daily living; IADL, instrumental activities of daily living; SD, standard deviation

**Fig. 2:**
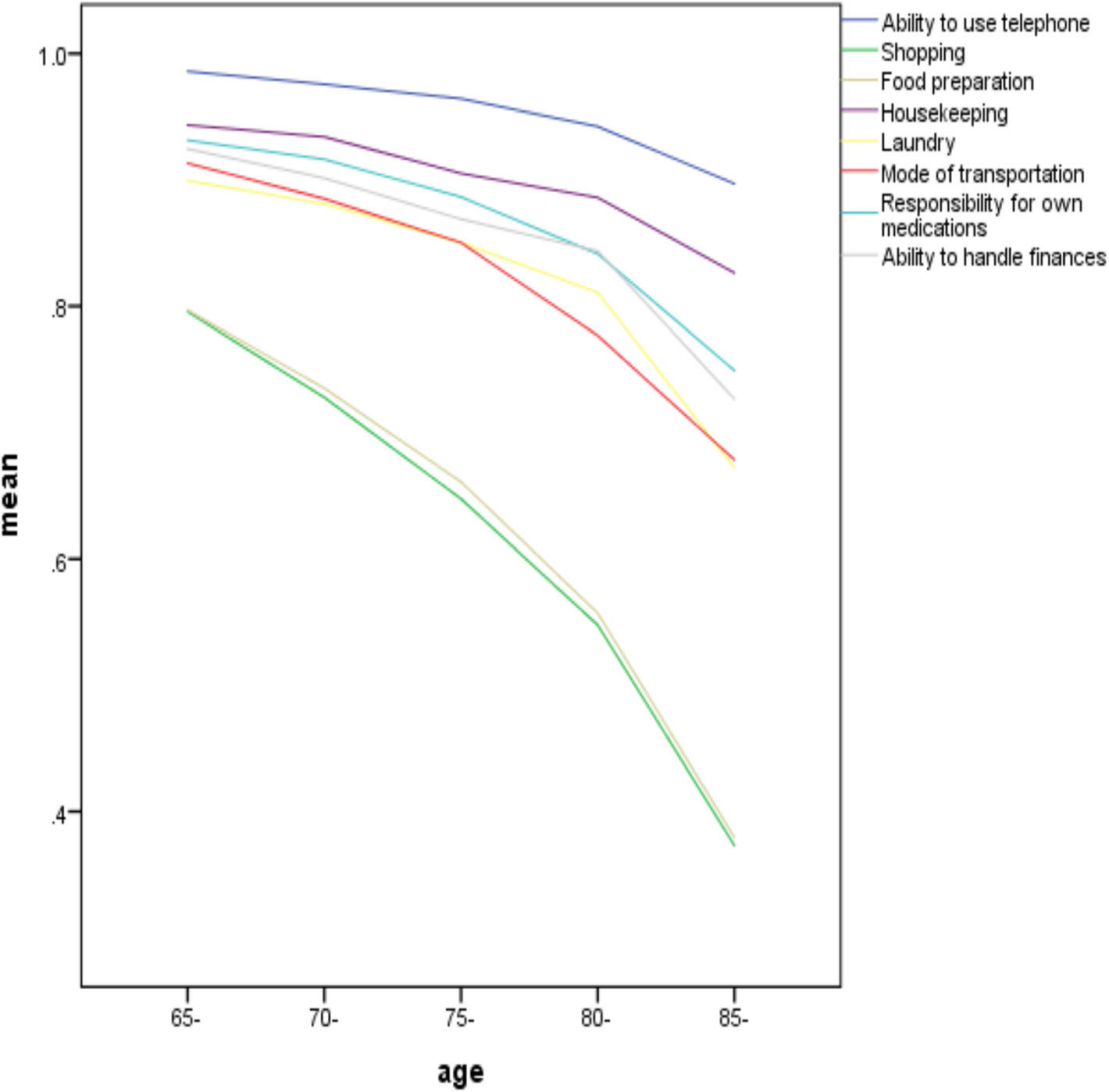
Risk of IADL by age. Abbreviations, IADL, instrumental activities of daily living

With regard to IADL, the mean IADL score was 6.76 ± 2.01. Shopping, food preparation, mode of transportation, doing the laundry, and ability to handle finances were limited for most inpatients in terms of IADL (see Fig. 2). A similar trend of decreasing scores alongside aging in the older population was also apparent in IADL. Likewise, there were significant differences among the IADL scores of each age group (*P* < 0.001).

### Associated factors of functional disability

As shown in Table 2, after controlling for the cluster effect of hospital wards, age was significantly associated with ADL. Compared with the 65–69 age group, the 70–74 age group (regression coefficient − 0.0163; 95% CI: − 0.0236, − 0.0090), 75–79 age group (− 0.0281; − 0.0361, − 0.0201), 80–84 age group (− 0.0674; − 0.0823, − 0.0525), and 85 and older age group (− 0.0909; − 0.1170, − 0.0648) were susceptible to ADL disability. Compared with participants of normal weight, emaciated participants were susceptible to poor ADL (− 0.0296; − 0.0460, − 0.0132). Aligning with univariate analysis results, frailty in participants (− 0.1459; − 0.1630, − 0.1288) and depression in participants (− 0.0473; − 0.0599, − 0.0347) were more likely to increase the risk of ADL disability in the multivariate model. Compared with outpatient department participants, those from emergency departments (− 0.1356; − 0.1750, − 0.0962) and transferred from other hospitals (− 0.0307; − 0.0551, − 0.0064) were susceptible to ADL disability. Former alcohol drinkers (− 0.0118; − 0.0227, − 0.0010) had an increased risk of ADL disability compared with nondrinkers. In addition, falling accidents in the past 12 months (− 0.0414; − 0.0567, − 0.0261), hearing dysfunction (− 0.0088; − 0.0176, − 0.0000), cognitive dysfunction (− 0.0305; − 0.0408, − 0.0202), urinary dysfunction (− 0.0420; − 0.0566, − 0.0275), and defecation dysfunction (− 0.0364; − 0.0479, − 0.0249) increased the risk of ADL disability. On the contrary, sex, ethnicity, educational level, smoking, living conditions, vision dysfunction, sleeping dysfunction, and marital status were not statistically associated with ADL disability.

**Table 2:** Factors associated with ADL from regression model

Characteristics	Univariate		Multivariate	
	Regression coefficient	95% CI	Regression coefficient	95% CI
Age				
65–69 years old (ref.)	–	–	–	–
70–74 years old	− 0.0205	− 0.0294, − 0.0115	− 0.0163	− 0.0236, − 0.0090
75–79 years old	− 0.0470	− 0.0577, − 0.0363	− 0.0281	− 0.0361, − 0.0201
80–84 years old	− 0.0981	− 0.1171, − 0.0791	− 0.0674	− 0.0823, − 0.0525
85 years old and above	− 0.1437	− 0.1786, − 0.1089	− 0.0909	− 0.1170, − 0.0648
BMI				
Obesity	0.0066	−0.0055, 0.0188	− 0.0032	− 0.0132, 0.0067
Overweight	0.0156	0.0081, 0.0231	0.0024	−0.0037, 0.0084
Emaciation	− 0.0626	−0.0813, − 0.0439	−0.0296	− 0.0460, − 0.0132
Normal (ref.)	–	–	–	–
Sex				
Male (ref.)	–	–	–	–
Female	−0.0091	−0.0167, − 0.0015	−0.0055	− 0.0130, 0.0019
Ethnicity				
Han (ref.)	–	–	–	–
Others	−0.0132	− 0.0283, 0.0020	0.0038	−0.0070, 0.0146
Educational level				
University	0.0298	0.0136, 0.0460	0.0067	−0.0072, 0.0206
Middle school	0.0309	0.0180, 0.0439	0.0024	−0.0085, 0.0134
Primary school	0.0227	0.0097, 0.0357	0.0059	−0.0041, 0.0158
Illiterate (ref.)	–	–	–	–
Marriage				
Divorced or widowed	− 0.0222	− 0.0358, − 0.0085	0.0089	− 0.0018, 0.0197
Married (ref.)	–	–	–	–
Frail				
Yes	−0.1888	− 0.2077, − 0.1698	−0.1459	− 0.1630, − 0.1288
No (ref.)	–	–	–	–
Depression				
Yes	−0.1058	−0.1214 -0.0903	− 0.0473	− 0.0599, − 0.0347
No (ref.)	–	–	–	–
Admission to hospital				
Emergency department	−0.1612	− 0.2031, − 0.1194	−0.1356	− 0.1750, − 0.0962
Outpatient department (ref.)	–	–	–	–
Transit from other hospitals	−0.0491	− 0.0788, − 0.0195	−0.0307	− 0.0551, − 0.0064
Others	−0.0028	− 0.0325, 0.0269	0.0088	− 0.0090, 0.0265
Living conditions				
Building with elevators (ref.)	–	–	–	–
Building without elevators	0.0028	−0.0064, 0.0120	0.0041	−0.0033, 0.0116
Bungalow	−0.0201	− 0.0327, − 0.0076	−0.0064	− 0.0176, 0.0048
Smoking				
Non-smoker (ref.)	–	–	–	–
Current smoker	0.0022	−0.0084, 0.0128	−0.0084	− 0.0180, 0.0013
Former smoker	−0.0058	− 0.0147, 0.0031	− 0.0038	− 0.0121, 0.0044
Alcohol drinking				
Non-drinker (ref.)	–	–	–	–
Current drinker	0.0154	0.0062, 0.0246	0.0002	−0.0083, 0.0087
Former drinker	−0.0102	− 0.0233, 0.0029	− 0.0118	− 0.0227, − 0.0010
Falling accidents in past 12 months				
Yes	− 0.0699	− 0.0878, − 0.0520	− 0.0414	− 0.0567, − 0.0261
No (ref.)	–	–	–	–
Vision				
Normal (ref.)	–	–	–	–
Dysfunction	− 0.0294	− 0.0401 -0.0188	− 0.0021	− 0.0115, 0.0074
Hearing				
Normal (ref.)	–	–	–	–
Dysfunction	−0.0330	− 0.0437, − 0.0222	−0.0088	− 0.0176, − 0.0000
Cognitive function				
Normal (ref.)	–	–	–	–
Dysfunction	−0.0659	−0.0796, − 0.0522	−0.0305	− 0.0408, − 0.0202
Sleeping				
Normal (ref.)	–	–	–	–
Dysfunction	−0.0282	−0.0361, − 0.0202	−0.0006	− 0.0071, 0.0059
Urinary function				
Normal (ref.)	–	–	–	–
Dysfunction	−0.0806	− 0.1014, − 0.0598	− 0.0420	− 0.0566, − 0.0275
Defecation function				
Normal (ref.)	–	–	–	–
Dysfunction	− 0.0802	− 0.0948, − 0.0656	−0.0364	− 0.0479, − 0.0249

Abbreviations, CI, confidence interval; BMI, body mass index; ADL, basic activities of daily living

As Table 3 shows, age was statistically associated with IADL disability. Compared with the 65–69 age group, the 70–74 age group (regression coefficient − 0.0235; 95% CI: − 0.0330, − 0.0141), 75–79 age group (− 0.0434; − 0.0565, − 0.0302), 80–84 age group (− 0.1040; − 0.1233, − 0.0848), and 85 and older age group (− 0.2166; − 0.2591, − 0.1740) were susceptible to IADL disability. Compared with participants of normal weight, emaciated participants were susceptible to poor IADL (− 0.0437; − 0.0678, − 0.0195). A higher level of education (0.0588; 0.0364, 0.0811) was statistically associated with a decline in the risk of IADL disability compared with illiterate participants. Frailty (− 0.2835; − 0.3159, − 0.2510) and depression (− 0.1491; − 0.1715, − 0.1268) in participants were more prone to increased risk of IADL disability in the multivariate model. Living in a building without elevators suggested a lower risk of IADL disability (0.0099; 0.0004, 0.0194) in the multivariate model, whereas there was no significant difference for participants living in a bungalow. Moreover, compared with nonsmokers, former smokers were prone to poor IADL (− 0.0142; − 0.0262, − 0.0022) whereas there was no significance among current smokers. Current alcohol drinkers (0.0176; 0.0062, 0.0290) had a reduced risk of IADL disability compared with nondrinkers, whereas there was no significance among former alcohol drinkers. Falling accidents in the past 12 months (− 0.0484; − 0.0641, − 0.0327), hearing dysfunction (− 0.0252; − 0.0413, − 0.0091), cognitive dysfunction (− 0.0742; − 0.0895, − 0.0588), urinary dysfunction (− 0.0365; − 0.0533, − 0.0197), and defecation dysfunction (− 0.0394; − 0.0559, − 0.0228) increased the risk of IADL disability. Sex, ethnicity, marital status, admission to hospital, vision dysfunction, and sleeping dysfunction were not statistically associated with IADL.

**Table 3:** Factors associated with IADL from regression model

Characteristics	Univariate		Multivariate	
	Regression coefficient	95% CI	Regression coefficient	95% CI
Age				
65–69 years old (ref.)	–	–	–	–
70–74 years old	−0.0312	− 0.0441, − 0.0183	−0.0235	− 0.0330, − 0.0141
75–79 years old	−0.0740	− 0.0908, − 0.0571	−0.0434	− 0.0565, − 0.0302
80–84 years old	− 0.1562	− 0.1821, − 0.1303	−0.1040	− 0.1233, − 0.0848
85 years old and above	− 0.3014	− 0.3572, − 0.2457	−0.2166	− 0.2591, − 0.1740
BMI				
Obesity	0.0135	− 0.0079, 0.0350	−0.0026	− 0.0192, 0.0139
Overweight	0.0275	0.0162 0.0388	0.0022	− 0.0059, 0.0104
Emaciation	−0.1123	− 0.1460, − 0.0787	− 0.0437	− 0.0678, − 0.0195
Normal (ref.)	–	–	–	–
Sex				
Male (ref.)	–	–	–	–
Female	−0.0233	− 0.0345, − 0.0122	−0.0021	− 0.0130, 0.0087
Ethnicity				
Han (ref.)	–	–	–	–
Others	−0.0504	−0.0832, − 0.0175	−0.0093	− 0.0316, 0.0130
Educational level				
University	0.1113	0.0847, 0.1380	0.0588	0.0364, 0.0811
Middle school	0.0993	0.0764, 0.1221	0.0395	0.0213, 0.0576
Primary school	0.0668	0.0424, 0.0913	0.0317	0.0130, 0.0503
Illiterate (ref.)	–	–	–	–
Marriage				
Divorced or widowed	−0.0583	−0.0816, −0.0350	−0.0013	− 0.0185, 0.0159
Married (ref.)	–	–	–	–
Frail				
Yes	−0.3713	−0.4060, − 0.3365	−0.2835	− 0.3159, − 0.2510
No (ref.)	–	–	–	–
Depression				
Yes	−0.2575	−0.2867, − 0.2284	−0.1491	− 0.1715, − 0.1268
No (ref.)	–	–	–	–
Admission to hospital				
Emergency department	−0.0591	−0.0859, − 0.0323	−0.0142	− 0.0348, 0.0064
Outpatient department (ref.)	–	–	–	–
Transit from other hospitals	−0.0085	−0.0452, 0.0283	0.0240	−0.0015, 0.0495
Others	−0.0150	− 0.0888, 0.0588	−0.0026	− 0.0500, 0.0447
Living conditions				
Building with elevators (ref.)	–	–	–	–
Building without elevators	0.0092	−0.0036, 0.0220	0.0099	0.0004, 0.0194
Bungalow	−0.0487	−0.0680, − 0.0295	−0.0137	− 0.0298, 0.0025
Smoking				
Non-smoker (ref.)	–	–	–	–
Current smoker	0.0245	0.0080, 0.0410	−0.0054	−0.0192, 0.0085
Former smoker	−0.0120	−0.0276, 0.0035	− 0.0142	−0.0262, − 0.0022
Alcohol drinking				
Non-drinker (ref.)	–	–	–	–
Current drinker	0.0563	0.0429, 0.0697	0.0176	0.0062, 0.0290
Former drinker	−0.0103	−0.0305, 0.0098	− 0.0092	−0.0242, 0.0059
Falling accidents in past 12 months				
Yes	−0.0984	−0.1180, − 0.0789	−0.0484	− 0.0641, − 0.0327
No (ref.)	–	–	–	–
Vision				
Normal (ref.)	–	–	–	–
Dysfunction	−0.0661	−0.0841, − 0.0481	−0.0132	− 0.0290, 0.0026
Hearing				
Normal (ref.)	–	–	–	–
Dysfunction	−0.0767	−0.0945, − 0.0589	−0.0252	− 0.0413, − 0.0091
Cognitive function				
Normal (ref.)	–	–	–	–
Dysfunction	−0.1517	−0.1733, − 0.1302	−0.0742	− 0.0895, − 0.0588
Sleeping				
Normal (ref.)	–	–	–	–
Dysfunction	−0.0529	−0.0634, − 0.0425	−0.0011	− 0.0094, 0.0073
Urinary function				
Normal (ref.)	–	–	–	–
Dysfunction	−0.0998	−0.1278, − 0.0718	−0.0365	− 0.0533, − 0.0197
Defecation function				
Normal (ref.)	–	–	–	–
Dysfunction	−0.1195	−0.1442, − 0.0948	−0.0394	− 0.0559, − 0.0228

Abbreviations, CI, confidence interval; BMI, body mass index; IADL, instrumental activities of daily living

In summary, sociodemographic characteristics (such as age), physical health variables (frailty, emaciation, hearing dysfunction, urinary dysfunction, defecation dysfunction, falling accidents in the past 12 months), and mental health variables (cognitive dysfunction, depression) were associated with both ADL disability and IADL disability. Patients from the emergency department or transferred from other hospitals and former alcohol drinking could increase the risk of ADL disability. Former smoking is a risk factor for IADL disability, whereas current drinking, higher-level education, and residing in a building without elevators were likely to maintain a better IADL performance.

## Discussion

This study systematically evaluated the level of ADL and IADL among different age groups as well as the potential factors associated with functional disability based on a hospital-based large-scale cross-sectional national survey in China. Application of a mixed-effect generalized linear model with the hospital as a random effect not only controlled the cluster effect of hospital wards but also examined the effect of potential associated factors, including indicators for sociodemographic characteristics, physical health variables, and mental health variables, on both the prevalence and extent of functional disability.

The average ADL score was 89.51 ± 19.29 and the mean IADL score 6.76 ± 2.01 for all participants in this study, which indicates older adults are low dependency upon functional status [5, 17]. Besides, the findings of our study reported that functional ability tends to deteriorate with the aging process [6, 25], differing from certain studies conducted in welfare institutions and among ethnic minorities [8, 9]. The average age of the elderly living in Japanese welfare institutions was (80.0 ± 7.4) years old [9], as a country with a serious aging process [4], the functional status of the 80 years age-group may differ from those of the same age in other countries. Also, most of the Yi ethnic minority resides in the southwestern parts of China. The differences in ADL and IADL that appear to exist between the geography, ethnicity, and target aging population, which require further study.

This study reveals another phenomenon worthy of attention. The average ADL score in our study is lower than the Yi ethnic minority, whereas IADL score is higher than them [8]. Perhaps because the inpatients enrolled in our study were all from tertiary hospitals and were more seriously ill when hospitalization, thus poor ADL ability was observed. However, low education level, outdated economy, healthcare, and culture contributed to the IADL problems in Yi ethnic minority older population [8]. Even though our government has made great efforts, and healthcare and economic conditions in ethnic minority areas have been improved, providing adequate geriatric care source, financial support, and social help among the elderly of ethnic minorities are warranted.

Our results suggested that with increasing age, changes in the ADL ability were mainly in walking up and down stairs, mobility, transfers (from bed to chair and back), toilet use, and bathing. The activities most impactful on IADL in the present study as graded from high to low were shopping, food preparation, mode of transportation, laundry, and ability to handle finances. This finding is consistent with those of previous studies reporting that functional status rapidly declines with increasing age in terms of speed and executive function with regard to [17, 26], for example, walking, household tasks, and shopping [25]. Although the biological aging process cannot be halted, it is urgent for our government to establish a home care-dominated, supported by community care, and supplemented with institutional care (such as welfare institution or nursing home) aimed at helping the elderly to self-manage their daily activities, reducing the risk of a decline in different aspects of functional independence, and meeting the escalating burden of the aging society [3, 27].

Apart from age, physical health variables such as emaciation, frailty bring about a series of negative effects on functional ability. Therefore, improving the nutritional status, healthy diet, and enough physical exercise is critical to inhibit the development and progression of poor functional status [28]. To temporizes or halt the disabled process, our government should develop a dietary nutrition plan in different age groups among the elderly and encourage and carry out physical exercise programs for older adults at the social level. Moreover, communities in the wide rural areas should regularly organize the elderly into morning exercise groups and dance groups, which can be easily spotted in China’s urban parks or street corners [29]. Also, vigorous evaluation studies in China on the health benefits of the dietary nutrition plan and regular physical activity programs among the elderly are required in the future.

Other physical health variables such as falling accidents in the previous 12 months, hearing dysfunction, urinary dysfunction, and defecation dysfunction were significantly associated with functional disability, which further supports previous research findings [8, 10, 11, 30]. Regarding these factors, early assessment, identification, and prevention are important. More advanced risk factor assessment scales and standardized nursing care measures may be useful in managing functional disability among older adults in the future [31].

With regard to mental health variables, our results suggested that functional disability was associated with depressive moods and cognitive dysfunction. This reminds us that ameliorate the depressive moods and delayed the deterioration of age-related cognition may help to further improve the functional ability [32]. Previous research suggested that depression in the elderly was associated with a lack of family support [33, 34]. Therefore, older adults may need more daily companionship from their family members, relatives, and friends, and geriatric care services, such as home care are urgently needed [33, 35]. For another thing, priority should be given to raising public awareness of cognitive dysfunction and increasing medical support resources for early prevention, evaluation, intervention, and medical treatment of mental illness and poor health status in the elderly [36, 37].

Older patients from the emergency department or other hospitals present a greater risk of ADL disability. Perhaps because the geriatric population in emergency departments are accompanied by immobility, failure to eat and drink, incontinence, and functional decline [38–40]. Older patients who are transferred from district hospitals or community hospitals probably only received basic inpatient services before [13], which may potentially increase the risk of poor ADL. Screening for ADL ability in emergency department patients and transferred patients are needed, which can inform prognosis and hospital services planning, target discharge planning, and post-discharge care.

Interestingly, former alcohol drinkers had an increased risk of ADL disability compared with nondrinkers, whereas current alcohol drinkers had a low risk of IADL disability. Perhaps because compared to small to moderate alcohol consumption, long-term and heavy alcohol consumption are at an increased risk of functional disability [41]. Also, a previous study indicated that older adults who consumed small to moderate amounts of alcohol were more likely to maintain functional status than nondrinkers, which might be related to the fact that small alcohol consumption has been associated with a decreased risk of cardiovascular events [10]. However, the detailed evaluation of alcohol consumption based on information about the quantity and frequency of drinking alcoholic beverages requires further research.

Our study indicated that former smokers had a higher risk of IADL disability, which further supported previous research findings [10, 42]. This indicates maintaining a vigorous lifestyle, such as tobacco-free, might be essential for improving functional status after decades. The government should continue to promote no-smoking policies in public areas and smokers should quit smoking as soon as possible [43].

Higher-level education and residing in a building without elevators were likely to maintain a better IADL performance, perhaps because well-educated participants could better comprehend the development of disease and maintain their physical function with a positive attitude [44]. In addition, residence in apartment block-type buildings in China is associated with better economic conditions. There exists the possibility that the geriatric population living in such buildings has a better quality of life and health monitoring that helps maintain IADL function.

There are limitations to this study that require discussion. First, because of convenience sampling, the patients enrolled in our study were selected from tertiary hospitals, and only one hospital in each province or municipality/city, which limited the generalizability of this study. Second, owing to the nature of the cross-sectional study design, we could only explore the relationship between functional disability and potential associated factors. Third, the target population in this study covered many wards or departments, and we did not analyze the medical treatment and nursing care use on functional disability in this study. Fourth, chronic diseases (such as cancer, diabetes, and cardiovascular diseases) could be associated with functional disabilities among older adults, the participants in this study covered many departments, and we did not analyze the impact of diseases in this paper. Prospective studies with more sophisticated evaluations are required in the future.

## Conclusions

This study suggests that poorer functional ability was associated with increasing age. Sociodemographic characteristics, physical health variables, and mental health variables were associated with functional disability. Investing adequate geriatric care sources, developing a home care-dominated, supported by community care, and supplemented with institutional care aimed at helping the elderly to self-manage their daily activities are warranted. In light of the factors associated with functional disability, attention should be paid to risk assessment, preventive measures, nutrition and physical activity intervention. These findings could have major importance for the planning of hospital services, discharge planning, and post-discharge care.

## Supplementary information

**Additional file 1 **Demographic characteristics of the participants (*N* = 9996). Abbreviations, BMI, body mass index; ICU, Intensive care unit.

## Data Availability

The datasets used and analyzed during the current study are available from the corresponding author on reasonable request.

## Abbreviations

BMI: body mass index
ICU: Intensive care unit
ADL: basic activities of daily living
IADL: instrumental activities of daily living
SD: standard deviation
CI: confidence interval
