# Supplementary material for: Potential associated factors of functional disability in Chinese older inpatients: a multicenter cross-sectional study
Source: BMC Geriatr. 2020 Sep 3;20:319. doi: 10.1186/s12877-020-01738-x (PMC7650523; doi:10.1186/s12877-020-01738-x)
Supplement: Supplementary file 1 — Additional file 1 Demographic characteristics of the participants (N = 9996). Abbreviations, BMI, body mass index; ICU, Intensive care unit. [file 12877_2020_1738_MOESM1_ESM.docx]

**Supplementary File 1.** Demographic characteristics of the participants (*N*=9,996).

| Variable | Number (n, 100%) |
| --- | --- |
| Sex |  |
| Male | 5778(57.80) |
| Female | 4218(42.20) |
| Age-group |  |
| 65-69 years old | 4234(42.36) |
| 70-74 years old | 2790(27.91) |
| 75-79 years old | 1753(17.54) |
| 80-84 years old | 884(8.84) |
| 85 years old and above | 335(3.35) |
| Education level |  |
| Illiterate | 1638(16.39) |
| Primary school | 2869(28.71) |
| Middle school | 4027(40.29) |
| University and above | 1460(14.61) |
| Marriage |  |
| Divorced or widowed | 1117(11.19) |
| Married | 8867(88.81) |
| Ethnicity |  |
| Han | 9412(94.16) |
| Minority | 584(5.84) |
| BMI |  |
| Obesity (≥28) | 991(10.07) |
| Overweight (24-27.9) | 3377(34.31) |
| Emaciation (<18.5) | 698(7.09) |
| Normal (18.5-23.9) | 4778(48.54) |
| Frailty |  |
| Yes | 1801(18.02) |
| No | 8195 (81.98) |
| Depression |  |
| Yes | 1771(17.72) |
| No | 8225(82.28) |
| Admission to hospital |  |
| Emergency department | 1319(13.20) |
| Outpatient department | 8284(82.87) |
| Transit from other hospitals | 329(3.29) |
| Others | 64(0.64) |
| Living conditions |  |
| Building with elevators | 3608(36.09) |
| Building without elevators | 4694(46.96) |
| Bungalow | 1694(16.95) |
| Smoking |  |
| Non-smoker | 6608(66.11) |
| Current smoker | 1114(11.14) |
| Former smoker | 2274(22.75) |
| Alcohol drinking |  |
| Non-drinker | 7647(76.50) |
| Current drinker | 1153(11.53) |
| Former drinker | 1196(11.96) |
| Falling accidents in past 12 months |  |
| Yes | 1422(14.23) |
| No | 8574(85.77) |
| Vision |  |
| Normal | 7794(77.97) |
| Dysfunction | 2202(22.03) |
| Hearing |  |
| Normal | 8057(80.60) |
| Dysfunction | 1939(19.40) |
| Cognitive function |  |
| Normal | 7469(79.43) |
| Dysfunction | 1934(20.57) |
| Sleeping |  |
| Normal | 5611(56.13) |
| Dysfunction | 4385(43.87) |
| Urinary function |  |
| Normal | 8596(85.99) |
| Dysfunction | 1400(14.11) |
| Defecation function |  |
| Normal | 8744(87.47) |
| Dysfunction | 1252(12.53) |
| Department |  |
| Surgical | 3296(32.97) |
| Medicine | 4694(46.96) |
| Neurology | 970(9.70) |
| Orthopedics | 719(7.19) |
| ICU | 317(3.17) |
| Province or municipality/city |  |
| Sichuan province | 1808(18.09) |
| Heilongjiang province | 1742(17.43) |
| Hubei province | 1824(18.25) |
| Beijing municipality/city | 1401(14.02) |
| Qinghai province | 1417(14.18) |
| Zhejiang province | 1804(18.05) |

Abbreviations, BMI, body mass index; ICU, Intensive care unit.
